# Ambulatory Typhoid

**Published:** 1907-08-31

**Authors:** 


### AMBULATORY TYPHOID.

Ambulatory typhoid fever is said to be very fatal, but it is clear that this is almost impossible of proof; a typhoid fever patient who never becomes ill enough to lie up in bed will not be diagnosed as typhoid fever at all; the commonest way for ambulatory typhoid to be recognised is by the patient dying, and the ulcerated ileum being found at the autopsy. It is clear that no one can say how many ambulatory cases have recovered, seeing that, being well enough to walk about all the time, they escape entirely from all statistics on the subject.

The fact that patients with typhoid fever may have no symptoms of it at all is well known. It emphasises the necessity for including enterica as a possibility in a great many obscure abdominal conditions that are atypical, and for not omitting to take serum for a Widal's test in other cases besides those in which the diagnosis of typhoid fever is fairly clear. The following is an instance of ambulatory typhoid:

A boy, aged 4½ years, was first regarded as ill two days before his death. Upon inquiry it was found that he had complained, a fortnight previously, of some "red places" on and below his knees, and of a little pain in his right ankle; he was kept in bed that day, but seemed nearly right the next. He continued well, and on the day but one before his death he ate a good dinner, and seemed to be in his usual health when put to bed. During that night, however, he began to have stomach-ache, was sick, and had some diarrhœa. The abdominal pain and vomiting persisted all night, and next morning there was a little blood with the stools. A doctor was sent for, and on arrival he found the patient pulseless, with dilated pupils, and short, shallow respirations. Nothing abnormal in the way of physical signs could be detected. Saline infusion and stimulants were resorted to, but without benefit. The child lived till the next day, nearly moribund all the while, and finally died, without any diagnosis having been made.

At the autopsy there was nothing abnormal to notice about the exterior of the body. The spleen was not enlarged; nothing was wrong except in the bowel. The lower part of the ileum was acutely inflamed; its outer surface, beneath the peritoneal coat, was covered by many hæmorrhages; the cæcum and first two inches of the colon were similarly affected. The mesentery was thickened as if by inflammatory exudation, and the glands in it were swollen and pale. The ileum, when opened, was found to be the seat of numerous typical "typhoid" ulcers affecting the Peyer's patches; many of the latter which were not actually ulcerated were swollen and inflamed. There were altogether 33 ulcers in the ileum, and in the cæcum and ascending colon there were some follicular ulcers in addition to general enlargement of the lymphoid follicles. Between the ulcers the mucous membrane of both ileum and colon was acutely congested, suggesting that the terminal symptoms were due to an acute entero-colitis superimposed upon the latent typhoid ulceration.

The condition, it might be urged, was possibly some other form of bowel trouble than enterica; in order to check this, however, some heart-blood was kept, and serum from it was used for a Widal's test. The latter was completely positive, clumping occurring well within half an hour when the serum was diluted 1 in 200.

